# Testing the effects of segmented crowdsource-selected messages to improve intentions to follow colorectal cancer screening recommendations: study protocol for a randomized controlled trial

**DOI:** 10.1186/s12889-026-26440-2

**Published:** 2026-01-31

**Authors:** Andy J. King, Yi Liao, Tianen Chen, Rohit Kanrar, Rumi Chunara, Drew Margolin, Dan Nettleton, Jeff Niederdeppe

**Affiliations:** 1 Department of Communication, University of Utah, 255 Central Campus Drive, Room 2400, Salt Lake City, UT 84112 USA; 2 Huntsman Cancer Institute, University of Utah, 2000 Circle of Hope Drive, Salt Lake City, UT 84112 USA; 3 Department of Communication, University of South Florida, 4202 E. Fowler Avenue, CIS1040, Tampa, FL 33620 USA; 4 Department of Statistics, Iowa State University, 2438 Osborn Dr., Snedecor Hall 1121, Ames, IA 50011 USA; 5 Department of Biostatistics, New York University, 708 Broadway, New York, NY 10003 USA; 6 Department of Computer Science & Engineering, New York University, 251 Mercer Street, Room 305, New York, NY 10012 USA; 7 Department of Communication, Cornell University, 237 Mann Drive, Mann Library 498B, Ithaca, NY 14853 USA; 8 Jeb E. Brooks School of Public Policy, Cornell University, 2301 Martha Van Rensselaer Hall, Ithaca, NY 14853 USA

**Keywords:** Health communication, Crowdsourcing, Colorectal cancer, Cancer screening, Health promotion, Cancer control, Health information

## Abstract

**Background:**

Colorectal cancer (CRC) is a leading cause of cancer-related morbidity and mortality in the United States (U.S.). CRC screening (CRCS) is an important method of cancer prevention and early detection, but often receives less public attention than other cancer and screening types. Regular CRCS is recommended for most individuals aged 45–75, though screening rates are lower than desired in the U.S. Despite similar screening rates, Black Americans experience a variety of CRC-related disparities including higher incidence and mortality rates in the United States. Research suggests Black Americans often do not receive timely follow ups to stool-based screening tests and receive lower quality endoscopic care. Effective public health communication strategies are needed to improve CRCS rates, particularly among populations experiencing disparities. The current study tests the ability of a novel approach to content generation and selection (crowdsourcing) to identify effective public health communication content.

**Methods/design:**

This protocol is for a five-arm, online, randomized controlled trial testing different CRCS content types among white and Black/African Americans. In total, 2,000 non-Hispanic Black (*n* = 1,000) and white (*n* = 1,000) Americans will be recruited into the trial, randomized into one of five conditions. The five conditions are based on content rankings and preferences from our previous work: (1) overall preferred content, (2) Black American preferred content, (3) white American preferred content, (4) median ranked content (i.e., “standard of care”), and (5) control/no exposure to content. The primary outcomes are intentions to adhere to CRCS recommendations and screening preferences for intentions to adhere. Secondary outcomes include likelihood of sharing information via social media and information sharing behavior. Comparisons will be examined for differences in outcomes as hypothesized, with exploratory analyses undertaken as well. The study is powered to detect a small effect, which is expected based on past research.

**Discussion:**

The trial aims to determine the effectiveness of crowdsource-selected CRCS messages to improve screening intentions and likelihood of sharing information via social media. This trial takes an important step in testing an innovative and scalable approach to identify and select content to be disseminated by public health communicators.

**Trial registration:**

NCT06712901.

**Supplementary Information:**

The online version contains supplementary material available at 10.1186/s12889-026-26440-2.

## Background

Colorectal cancer (CRC) is one of the most diagnosed and deadliest cancers in the United States (U.S.) [1]. This year, over 150,000 people are expected to be diagnosed with CRC, and over 50,000 people are expected to die from CRC [1, 2]. CRC screening (CRCS) serves as a tool for prevention and early detection, but tends to receive less public attention than other cancer topics and screening tests [3–7]. The United States Preventive Services Task Force recommends CRCS for individuals between the ages of 45 and 75 [8]. Recommendations include one of the following: completion of a stool test (e.g., fecal immunochemical test or fecal occult blood test) annually, a flexible sigmoidoscopy every five years, or a colonoscopy every 10 years depending on results and discussions with primary care physicians [8]. The U.S. public health goal (e.g., Healthy People 2030) for CRCS is to have screening rates for adults ages 45–75 reach 68.3% by 2030, though estimates are currently below that goal (58.7%) [9]. CRCS can improve CRC outcomes and offers an important outlet for both cancer prevention and early detection.

CRC incidence and mortality rates are highest among Black Americans, along with American Indian and Alaskan Native populations, compared to non-Hispanic White Americans [1]. While some CRC-related disparities have decreased due in part to increased CRCS rates across all racial groups, racial disparities between Black and White Americans persist in CRC incidence, mortality, and stage of diagnosis [1, 2]. Overall screening rates are similar for Black and White individuals in the United States [1], but Black Americans are less likely to receive high-quality colonoscopies [10] and timely follow-up to certain screening tests (e.g., stool tests) [11].

Like most health issues, complicated multi-level factors contribute to disparities in, and barriers to, CRCS quality and increased uptake for Black Americans. Individual factors include limited knowledge about benefits of CRCS, low perceived personal risk, feelings of fear and anxiety about potential diagnoses, cost of screening, medical mistrust, lack of interest in CRCS, and concerns about invasiveness and intrusiveness of CRCS [12–15]. Systemic factors include inequalities in access to cancer screening information, structural racism, limited continuity of care and lack of a physician recommendation about screening [15–17]. While the factors influencing barriers to increasing CRCS rates are complex, incomplete or misunderstood information about risk and screening procedures is a prominent modifiable factor influencing screening rates.

There is a critical need to identify approaches to communicate effectively about CRCS to eligible screening subpopulations—particularly among Black Americans (who have highest rates of CRC incidence and mortality and are the second largest racial group in the US) and White Americans (who by virtue of population size experience the highest number of colorectal cancer diagnoses and deaths). Public health communicators have continuing concerns about information quality of health content on social and digital media [18–22], including cancer information specifically [23–25], with evidence that inaccurate information spreads more widely and easily than accurate information [26]. We lack evidence-based guidance on effective CRC screening messaging via digital and social media platforms to increase rates of CRC screening among Black and White Americans.

### Curating and crowdsourcing content for the trial

Current guidance for public health communication content development is to engage in theoretically grounded approaches that encourage the use of behavior change and information processing theories [27]. The challenge with this approach is that research suggests there are small effects of modifiable message features [28], with single messages often addressing multiple theoretical constructs, which suggests the need for innovative ways to monitor the public communication environment (PCE) for strategies and content approaches that could work, as well as novel methods for identifying content likely to influence and engage intended audiences. Developing and testing strategies to curate and disseminate accurate and effective content about CRCS that people want to share is a priority for public health communicators. In service of that priority, we engaged a multi-step process to identify, curate, and crowdsource content to test in the current study.

The initial step of the process was to monitor the PCE, using computational approaches, to determine what people and organizations were saying about CRCS. This process required the scraping of content from social media sites (e.g., Twitter/X and YouTube) [29–31] and evaluating other digital/online content (e.g., comprehensive cancer center websites; popular health sites) to identify appeals and strategies for communicating about CRCS. Our team, all with expertise in health communication, then engaged in qualitative evaluation of thousands of messages to identify messages that seemed unique from others. This approach, described in more detail below, provided us with dozens of distinct messages to test.

This approach to monitoring the PCE provides useful insights, and a large corpus of content to consider, but comes with several limitations (e.g., not everyone uses social media and not everyone might post what they want publicly). Combining computational methods of monitoring the PCE with crowdsourcing methods to evaluate audience preferences can help address some of those limitations. Crowdsourcing refers to a “distributed problem-solving and production model that leverages the collective intelligence of online communities to serve specific organizational goals” [32], which can occur online or offline [33]. Crowdsourced data function as an important source of knowledge about improving public health [34] and have been suggested as an outlet to improve decision making related to cancer self-examinations [35]. Communication researchers have adopted an approach to using crowdsourcing in message selection, which is referred to as a wiki survey [36, 37]. Wiki surveys offer researchers the opportunity to evaluate and generate messages on topics of interest by presenting a pair of messages and asking people to choose which message they believe is a more effective message. Participants are allowed to choose a message and add their own content if they have an idea to provide. If researchers determine that the participant-generated content is unique to the set of messages being tested, those messages can be added to the pool being tested and selected as preferred.

Crowdsourcing approaches to content curation and creation, like wiki surveys, have clear advantages to more traditional approaches like focus groups (small samples, limited ability to test received feedback) or surveys (small samples of participants and message evaluations). The wiki survey allows for layperson-generated content to be incorporated into message selection processes. This has considerable value because it allows for people unfamiliar with message content to put forward messages that resonate with them, rather than relying on content developed by experts or advocates for a particular topic. By accessing interpretations, preferences, and creativity from the crowd, there is considerable potential to identify new message content categories and strategies. Additionally, the wiki survey has the ability to integrate that participant generated content into the overall crowdsourced evaluation task. The product of the wiki survey is a ranking of messages, content pre-selected by researchers *and* generated by participants, that theoretically corresponds to what messages are most likely to be effective. Past research, however, has not directly tested if highly ranked messages selected through wiki survey approaches are actually more effective when tested in a randomized trial. In general there have been and continue to be debates on the best way to identify and select, a priori, effective public health communication content [38]. The current trial addresses this gap in the literature and tests if a crowdsourcing approach is an effective path to determining what CRCS content should be disseminated widely to improve CRCS-related outcomes (e.g., following screening recommendations and sharing accurate information).

### Overall aim and specific hypotheses/research questions

The current study is the last of several steps in testing a comprehensive approach to monitoring the PCE (described above), gaining feedback about existing online and participant-generated content, and determining if crowdsourcing can improve the selection of effective health messages to influence key outcomes including intentions to follow screening recommendations and likelihood of sharing messages on social media. The current study evaluates if the crowdsourced approach is a valid way to identify effective messages that can be scaled and used to promote CRCS among diverse populations. We will test the effects of messages in a five-condition randomized trial, with a recruitment goal (*N* = 2,000) oversampling non-Hispanic Black Americans (*n* = 1,000). The goal of the trial is to test the hypotheses and answer the research questions posed below.

- H1: Exposure to any message will result in (a) higher CRCS intentions and (b) greater likelihood to share CRCS information compared to the no exposure control condition.
- H2: Messages identified as being preferred overall will result in (a) higher CRCS intentions and (b) greater likelihood to share information via social media compared to median-ranked messages.
- H3: Messages identified as being stronger by a specific subpopulation (e.g., Black Americans or White Americans, respectively) will result in (a) higher CRCS intentions and (b) greater likelihood to share CRCS information compared to those outside of that subpopulation receiving the same messages.
- RQ1: How does self-reported race category interact with message condition to influence (a) higher CRCS intentions and (b) greater likelihood to share CRCS information?
- RQ2: Does the crowdsource-ranking approach have validity in identifying effective CRCS messages for intended audiences?

## Methods/design

### Study design and trial registration

**Fig. 1:**
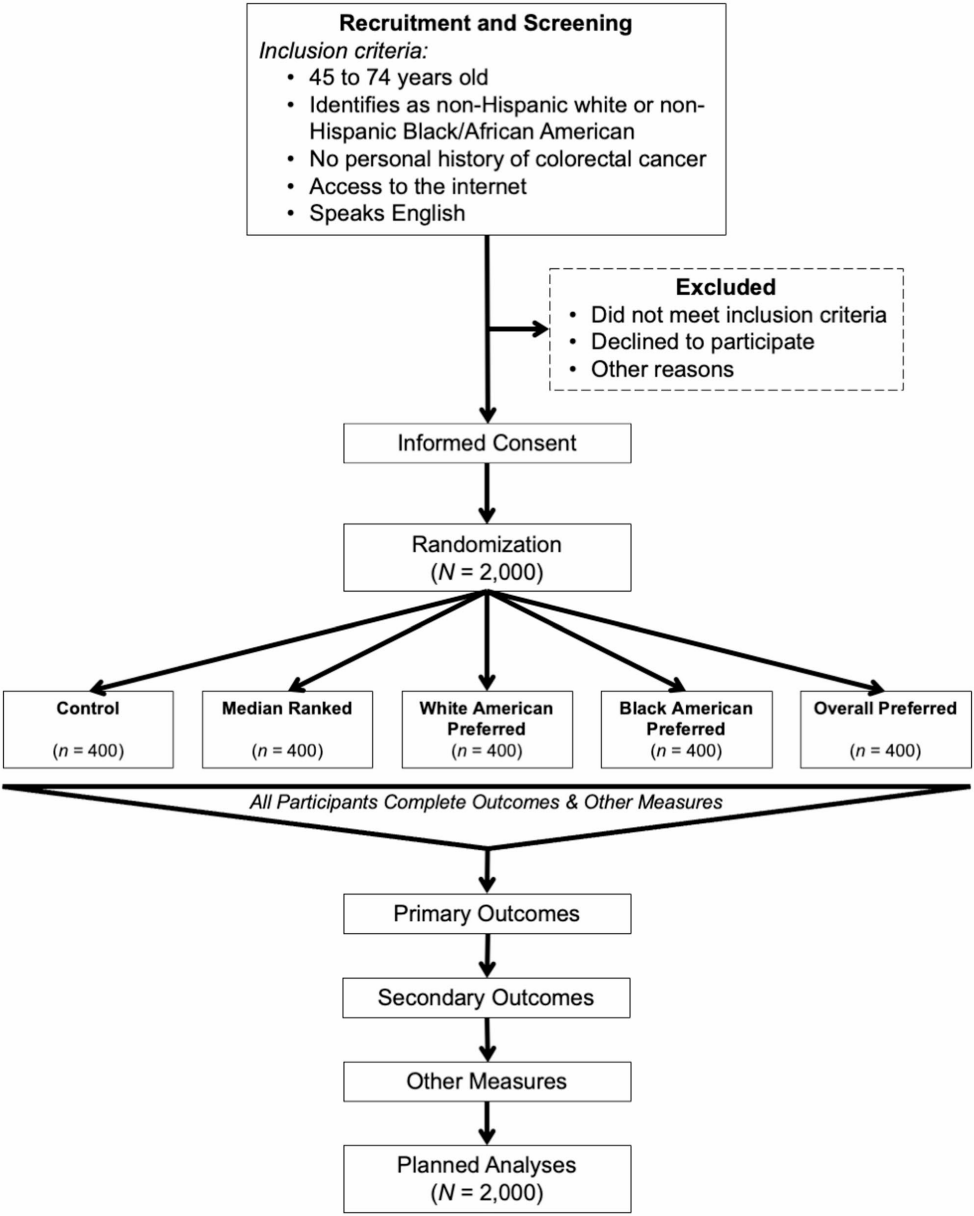
*Flow chart representing the study design*

We are conducting an online randomized controlled trial, with a five-group between-subjects post-test design (see Fig. 1). This protocol has been registered on ClinicalTrials.gov (NCT06712901) on 2nd December 2024. The study will begin recruiting participants in mid-December 2024, with recruitment expected to be completed in January 2025. This protocol submission follows the SPIRIT checklist [39] and this version is the most recent (Version 4, submitted 18th November 2025).

### Participant recruitment and inclusion criteria

Participants will be recruited online by the National Opinion Research Center (NORC). The study will use two sampling approaches to ensure sufficient recruitment for the study. The probability sample for the study will be selected from NORC’s AmeriSpeak Panel, with that sample being supplemented by a nonprobability sample arranged and vetted by NORC.

To participate in the study, potential participants must identify as non-Hispanic white or non-Hispanic Black/African American, live in the United States, speak English, have no history of colorectal cancer, and be aged 45 to 74 years old. Participants will be excluded from analysis if they complete the study in less than one-third the median completion duration, skip more than 50% of eligible study items, or straight line responses. Participants will receive an incentive from NORC for their participation in line with preexisting agreements equivalent to approximately $2 USD. The study is expected to take around 15 min to complete. Data provided by NORC for analysis will be de-identified and not able to be connected to participants.

### Sample size calculation

We assume a small effect size, consistent with past research on health communication messaging [28], in calculating the size of our sample. We use general effect size estimates from past work because there is not a trial testing this exact approach to messaging about colorectal cancer screening. Using a power calculation program [40] to determine sample size for F tests, assuming a small effect (Cohen’s *f* = 0.10), power of 0.90, alpha at 0.05, with 10 groups (five conditions and two distinct racial groups) and the inclusion of covariates, a total sample size of 1,992 is required. We aim to recruit 2,000 participants to ensure adequate power and allow for between-group comparisons with equal group sizes.

### Study procedure, randomization, and study conditions

**Fig. 2:**
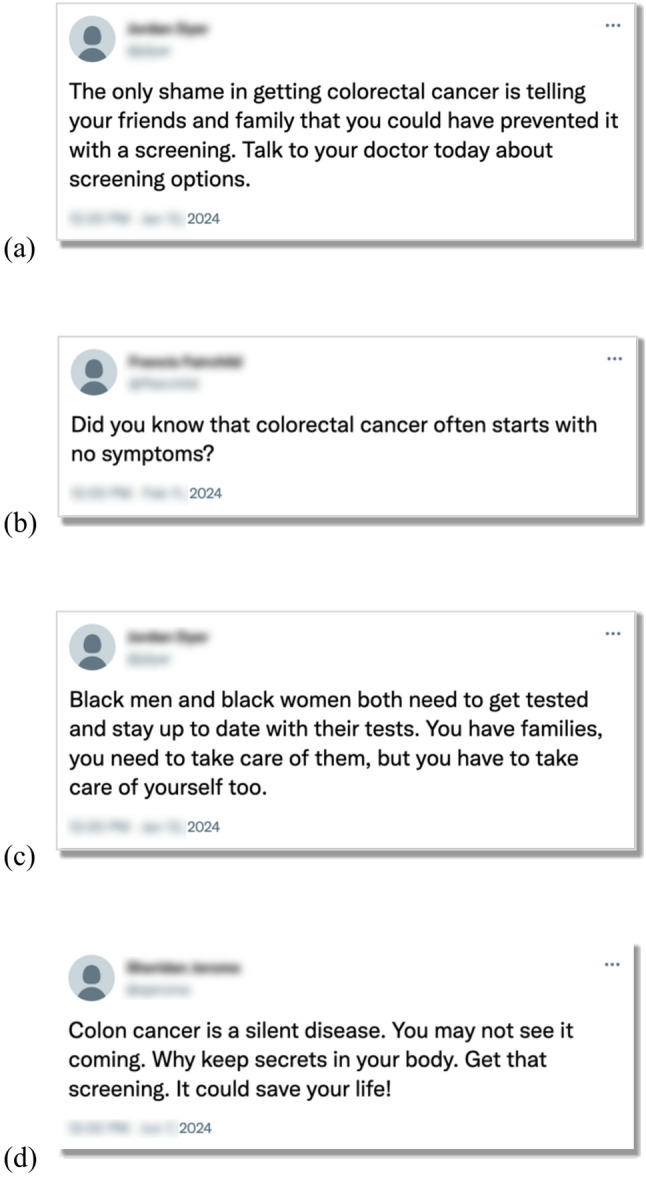
*Examples of information presentation in the four experimental conditions: (****a***) *median ranked, (****b****) White American preferred, (****c****) Black American preferred, and (****d****) overall preferred*

NORC will contact panel members about their interest in participation. Interested members will click a link that screens them for the inclusion criteria. If members meet the inclusion criteria, the online system provides informed consent information. Panel members consenting to participate move forward with the study and are randomized into one of the five study conditions. A least filled quota approach will be used along with randomization to ensure even numbers of participants across the five study conditions. Study conditions correspond to the categories of CRCS messages participants will receive, though the control (no exposure) group will not receive any CRCS messages. Messages in each of the four study conditions (see Table 1; seven messages per condition) will be presented to participants in a random order, with messages formatted to look like social media messages (see Fig. 2).

**Table 1:** *Information presented to participants by condition*

***Median Ranked***	***Black American Preferred***
• If you are 45 + you need to get your butt to the doctor. Colorectal cancer is the 2nd leading cause of cancer deaths among men and women combined in the U.S. But screenings are easy + save lives. Just ask Jimmy Kimmel!	• 1 in 2 men get cancer in their lifetime. Women? 1 in 3. Black men: 47% higher chance of dying from colon cancer than White men. Let’s change the stats. Help us prevent cancer by encouraging brothers (ages 45–75) to get screened.
• Each life has beauty and value! Be strong and get screened for colon cancer. Don’t let anything prevent you from your health care journey. Your life is worth it.	• It’s time to get screened. Anyone can get colorectal cancer, but do you know that Black people have the highest rate among all races/ethnicities in the US? If you’re African American and 45 years old, talk to your doctor!
• If undetected colon cancer could be deadly.	• African Americans are at higher risk for colon cancer. According to the American Cancer Society, African Americans are about 20% more likely to get colorectal cancer and about 40% more likely to die from it than most other groups. Please go get a colonoscopy if you are of age. Please.
• This disease knows no color or gender it’s a killing machine on a mission of death let’s do what we can to stop this disease dead in its tracks.	• For everyone, especially BLACK PEOPLE, have a COLONOSCOPY. AND BLACK MEN, THERE IS NO SHAME! HAVE A PROSTATE EXAM!
• Large polyp found and removed in routine colonoscopy for occult gastrointestinal (GI) bleed. Colon cancer was prevented in this case. Colorectal cancer is a common and lethal disease, but can be prevented. Get screened if you are 45 years old. Some patients should start screening earlier.	• Colorectal cancer (CRC) death rates have been significantly higher in Black men than in White men since 2005. Due to COVID-19, colonoscopy screening for colon cancer declined nearly 90%. We need to raise awareness and encourage preventative screenings.
• More than 55% of new colorectal cancer cases could be prevented by better lifestyle choices. Healthy lifestyles and greater public investment in prevention and citizen education is key.	• According to the American Cancer Society, with colon cancer, Black people and people of color have a 20% higher incidence rate and 40% higher mortality rate than White people do.
• The only shame in getting colorectal cancer is telling your friends and family that you could have prevented it with a screening. Talk to your doctor about screening options.	• Black men and black women both need to get tested and stay up to date on their tests. You have families, you need to take care of them, but you have to take care of yourself too.
**Overall Preferred**	**White American Preferred**
• Do you know screening is the No. 1 way to prevent colorectal cancer, the second deadliest cancer in men and women combined? Screening can help detect cancer early, when it’s still highly treatable. It’s safe and it saves lives! Schedule your screening today.	• One colon cancer death every 10 min. Screening prevents colorectal cancer.
• Colon cancer is a silent disease. You may not see it coming. Why keep secrets in your body. Get that screening. It could save your life!	• Public service announcement for the day…don’t be afraid to get your routine colonoscopy! Just had mine. All is good! Not gonna lie the prep you need to drink is nasty, but the anesthesia is glorious! It really is a piece of cake and worth it to get checked.
• It doesn’t matter who you are, colorectal cancer can affect anyone at any time. It does not discriminate.	• Hand in hand with colorectal cancer prevention is screening- have you spoken to your primary care provider yet about your options?
• Colon cancer is one of the most preventable cancers, but only if you get screened. Now, there are more screening options than ever, from at-home test kits to the traditional colonoscopy.	• Screening is much easier than chemotherapy and surgery.
• Colonoscopy is the gold standard for screening. If anything is found, the doctor can take a biopsy or remove the polyps right away. If everything checks out without any concerns, you are good to go for many years.	• Did you know that colorectal cancer often starts with no symptoms?
• Even if you think you are living a healthy lifestyle, cancer screenings are still a valuable tool for your health. Talk it over with your doctor today.	• Rising rates of colorectal cancers among younger adults have prompted a U.S. task force to lower the age to begin screenings to 45, rather than the current age of 50
• Getting routine screenings is imperative, regardless of family history. It’s worth it to have peace of mind that you don’t have colorectal cancer.	• If you think that you can wait until you’re older to be tested for colon cancer, it could be the biggest mistake you ever made in your life.

NOTE: See Fig. 2 for how messages appear to participants. Messages appear in a random order. Messages selected based on rankings by participants in formative research. Control (no exposure) condition receives no messages

We selected messages for each study condition based on the findings from a previous study [41]. In that study, participants with the same inclusion criteria viewed 30 pairs of messages from an initial pool of 68 messages. These messages were curated by our research team based on thorough monitoring of social and digital media content from various platforms and websites. Participants viewed pairs of messages and selected the message they believed was a better message. We used a crowdsourced approach called a wiki survey [36, 37], where participants also had the option to add their own message content if they had an idea for different content. Halfway through the completion of the wiki survey, our team examined participant-generated content and added an additional 25 messages to the pool to be evaluated by participants for a total of 93 messages. After receiving ranking data from all participants (over 60,000 rankings), our team used a Bayesian modeling and analysis approach (full results to be reported in a future publication) to determine the ranked preferences of message content overall, by Black Americans specifically, and by White Americans specifically. We then examined these rankings to create conditions based on messages that define our conditions (see Table 1). These study conditions are based on messages that were overall preferred, preferred by Black Americans, preferred by White Americans, and those that were median ranked (e.g., ranked near the midpoint by both groups). We did not include messages that were ranked low by participants, as disseminating low quality messages would never be the intentional practice of public health communicators. Instead, we conceptualized median-ranked items as akin to a “standard of care” for messages in the public communication environment. We include a true control (no exposure) group to test whether any messaging is better than none. Participation is expected to require median completion time of 15 to 20 min.

### Study outcomes

All measures, including wording for primary and secondary outcomes, appear in Table 2.

**Table 2:** *Primary outcomes*,* secondary outcomes*,* and list of other questionnaire measures*

***Primary Outcomes***	
• Intentions to adhere to colorectal cancer screening (CRCS) recommendations	
Many medical organizations and professionals recommend colorectal cancer screening for most people from ages 45 to 75. Recommendations include a variety of different exams like stool-based tests that you would do every year, or endoscopic methods like a sigmoidoscopy (every 5 years) or colonoscopy (every 10 years).	
Stool-based tests include options like a fecal occult blood test (FOBT) or fecal immunochemical test (FIT) that are take-home, non-invasive exams in which you send a sample of your stool to a lab to determine if you have signs of cancer or other health problems. Sigmoidoscopy and colonoscopy are exams in which a tubular scoping device is inserted in the rectum to view the colon for signs of cancer or other health problems.	
Given these recommendations, please indicate your response to the following question: How likely is it that you will follow one of the screening recommendations as outlined above?	
**Response options:** Very unlikely (1), unlikely, neither unlikely nor likely, likely, very likely (5)	
• Screening preferences for intention to adhereIf you followed the recommendations as listed above, which category of screening method would you be most likely to choose?	
**Response options (choose one):** stool-based tests (e.g., FOBT or FIT); endoscopic/scoping approach (e.g., colonoscopy or sigmoidoscopy); whatever my doctor recommends to me; something other than a stool-based or scoping approach; I would not follow the recommendations	
***Secondary Outcome***	
• *Likelihood of sharing information via social media*	
How likely is it that you will share any of the messages you just read about colorectal cancer screening? (only for experimental conditions; control condition item: How likely is it that you will share any information about colorectal cancer screening? )	
**Response options**: Very unlikely (1), unlikely, neither unlikely nor likely, likely, very likely (5)	
• *Information sharing behavior*	
Participants will be classified based on their response to one item and if they click on a link later in the survey. The item posed to participants will say, “Would you like to receive information on colorectal cancer screening to share with friends, family, or via social media? If you click yes here, we will provide you a link to more information at the end of the survey.	
”**Response options**: Yes/No	
***Other Questionnaire Measures***	
• *Previous CRCS behavior*	• Information seeking click behavior
• *CRCS risk perceptions*	• *CRCS norms*
• *CRCS self-efficacy*	• *Perceived racism in healthcare*
• *CRCS response efficacy*	• *Social media perceptions*
• *Policy support related to CRCS equity*	• *Social media use*
• *Perceived disparities in CRC*	• *Homophobia*
• *Perceived prevalence/norms of CRCS*	• *Insurance status*
• *Cancer fatalism/overload/confusion*	• *CRC family history*
	• *Demographics*

#### Primary outcomes

The primary outcomes are (1) intentions to adhere to recommendations for CRCS in the future and (2) screening preferences related to intentions to adhere. Additionally, we will evaluate people’s intentions to adhere test preference to better understand if preferences for CRCS options differ. Wording and response options for the primary outcomes are provided in full in Table 2.

#### Secondary outcome

The secondary outcomes are (1) likelihood of sharing information via social media and (2) information sharing behavior. Likelihood of sharing information will be evaluated by a single item that asks the four study conditions if they would share any of the messages they just viewed, whereas the control condition will be asked if they intend to share any information about CRCS in general. For information sharing behavior, participants will respond to a yes/no question if they want information to share via social media. Wording and response options appear in full in Table 2.

#### Other measures

Participants will also complete a variety of other measures, listed in Table 2, that will be examined in exploratory analyses in relation to and independent of primary and secondary outcomes. Full wording and details about these items will be included with de-identified data shared via OSF on the timeline noted in the previous section.

### Planned analyses

We will use SPSS, Stata, or R to perform analyses related to hypotheses and research questions related to our primary and secondary outcomes. We will examine mean comparisons between the five conditions and participant race (respondents identifying as white or Black Americans), as well as the interaction, specific to intentions to adhere to recommendations for CRCS and likelihood of sharing information via social media. Regression analyses will be used to examine secondary outcomes. We do not have predefined plans for analyses of non-primary and non-secondary measures.

### Ethical approval and planned data sharing

This study has been reviewed and determined exempt from continuing review by the University of Utah institutional review board (IRB_00183491). We will share all de-identified data, via the Open Science Framework (OSF), after publication of results related to the analyses of primary and secondary outcomes. This is a minimal risk study, so no additional data monitoring committee is required.

## Discussion

This study is a randomized controlled trial of the short-term effects of CRCS information content, which have potential long-term implications for improving CRCS rates among populations experiencing CRC-related disparities. We designed the trial to maximize internal validity, including powering the study to examine differences on our specific primary and secondary outcomes. The trial uses a blend of content selected through a novel approach to determine if such approaches can be useful for public health communicators. More specifically, this trial represents the first test of the ability of crowdsourcing approaches (e.g., wiki surveys) to identify effective content for public cancer communication efforts. Results of the trial will provide initial evidence about the utility of these novel approaches to identify and select culturally preferred, effective, and audience-centered content.

While the study is properly powered to identify differences in primary and secondary outcomes, there are still limitations that merit note. For example, there are often small effects based on a priori content categories in health communication research, so the messages selected—even though they are several in number, randomly presented, and audience-preferred—may not produce short-term differences in the different study arms. Long-term behavioral follow-up of participants is not possible given the scope of the project, so the trial is unable to provide evidence about the long-term, enduring, or unintended effects of the message selection approach.

The trial takes an important step in innovating scalable approaches to identify and select content to be disseminated by public health communicators. Better understanding how to engage in overarching communication strategizing for public health efforts is an important component of developing multi-level system solutions.

## Supplementary Information

Supplementary Material 1.

Supplementary Material 2.

## Data Availability

Data sharing is not applicable to this article as no datasets were generated or analyzed for the protocol paper.
