# Supplementary material for: Testing the effects of segmented crowdsource-selected messages to improve intentions to follow colorectal cancer screening recommendations: study protocol for a randomized controlled trial
Source: BMC Public Health. 2026 Jan 31;26:755. doi: 10.1186/s12889-026-26440-2 (PMC12947398; doi:10.1186/s12889-026-26440-2)
Supplement: Supplementary file 1 — Supplementary Material 1. [file 12889_2026_26440_MOESM1_ESM.pdf]

Figure Supplement. SPIRIT Figure.

| | STUDY PERIOD | | |
|---|---|---|---|
| | Pre-Study | Randomization | Post-Randomization |
| **TIMEPOINT** | $-t_1$ | 0 | $t_1$ |
| **ENROLLMENT (See Figure 1):** | | | |
| **Eligibility screen** | X | | |
| **Informed consent** | X | | |
| **Randomization to Intervention** | | X | |
| **INTERVENTIONS (See Table 1):** | | | |
| ***Control (No Exposure)*** | | | X |
| ***Median Ranked*** | | | X |
| ***Black American Preferred*** | | | X |
| ***White American Preferred*** | | | X |
| ***Overall Preferred*** | | | X |
| **ASSESSMENTS (See Table 2):** | | | |
| ***Primary Outcomes*** | | | X |
| ***Secondary Outcomes*** | | | X |
| ***Other Measures*** | | | X |
